# Pharmacy students' anxiety towards research during their undergraduate degree; How to reduce it?

**DOI:** 10.1371/journal.pone.0176095

**Published:** 2017-04-18

**Authors:** Mari Kannan Maharajan, Kingston Rajiah, Ai May Tam, Siew Ling Chaw, May Jing Ang, Mei Wan Yong

**Affiliations:** 1 Department of Pharmacy Practice, School of Pharmacy, International Medical University, Kuala Lumpur, Malaysia; 2 BPharm (Hons.,) graduate, School of Pharmacy, International Medical University, Kuala Lumpur, Malaysia; University of Westminster, UNITED KINGDOM

## Abstract

**Objective:**

To measure pharmacy students' anxiety towards research and how academic support, academic effort, attitude and self-efficacy influence their research anxiety.

**Methods:**

A cross-sectional study was conducted with undergraduate final year students of pharmacy using a convenient sampling method. A validated self-administered questionnaire was used.

**Results:**

Response rate for this study was 85.9% (128 students from a population of 149). The participants agreed that they read literature to understand research, but did not attend research-related coursework. Most participants (91.4%) felt that they were under stress while doing research. Almost all participants (97.6%) felt that they were doing very badly during their data analysis or they may fail their research projects. The majority of participants agreed that help from the lecturers' and friends in research give emotional support for their research activities.

**Conclusion:**

Academic support for pharmacy students, along with their additional academic effort will improve the students' self-efficacy and reduce research anxiety.

## Introduction

The changes in modern health care are rapid and the pharmacy profession is undergoing an evolution to meet the changing health care needs around the world. The transformation of healthcare practice always based on medical and health related research [1, 2]. The research in pharmacy focusing more towards evidence based medicine, which is a key to modern healthcare. Considering a broad and multidisciplinary aspects of drug therapy and outcome, there is a need for pharmacy and pharmacy practice based research to improve patient care [3]. In spite of pharmacists being aware of the importance of practice based research, they are less likely to participate in research themselves [4–6]. There is a need for pharmacists to view research participation as part of routine care [7].

Developing a research-oriented pharmacy graduate, with the right attitude towards research is one of the fundamental goals of educational systems [8]. Pharmacy schools around the world have different methods of engaging their students in scholarly research activities [8–11]. Involvement of students in research and research-related coursework is one of the widely used methods to engage students. Faculty observations reveal that, developing positive attitudes towards research among undergraduate students is one of the major challenges globally [12]. There are a number of factors being reported for influencing students’ attitude towards research.

The academic support from the lecturers or research supervisors may contribute students’ high satisfaction levels. A lecturer’s role in mentoring, identifying problematic areas in research and implementing necessary revisions during the research is essential to succeed in the research project. During research projects, assistance from the supervisors prevent academic problems for students. Somers et al., (2004) reported that academic mentoring assist to improve young students’ academic functioning [13]. Academic supports such as tutorial classes, direct mentoring and skills program helps in students’ grades [14]. Aslam et al., (2005) reported that a mentorship by lecturer plays an important role in student research [15]. However, to date, there is no study has investigated direct link between academic support and undergraduate students’ research anxiety.

Generally, students’ academic performance in any area is interrelated with their effort [16–19]. However, there were very limited literatures provide more details on how academic effort by students influences their interest in research. Therefore, this study focused academic efforts of students towards research

In practice, research anxiety acts as a carrier filter, preventing fresh graduates from entering into research related jobs. Anxiety significantly affects students’ learning and academic performance during their study [20]; more importantly, it may obstruct young adults’ academic achievements [21]. It was also reported that students’ research anxiety and doubts can greatly affect their ability to master research concepts [22]. Though anxiety may be help in the learning process to some extent, optimum performance is affected by high levels of anxiety [23]. However, undergraduate students usually tend to view research as difficult and stressful and some even develop a “phobia’ towards research [24, 25].

Self-efficacy beliefs are essential for professional students, as they one of the key predictors of academic performance [26, 27]. Self-efficacy in leading a research is vital for an individual to succeed in research-related field. “Research self-efficacy” can predict the students’ interest in the research field [28]. Many studies have shown that “academic support” was believed to improve students’ productivity and help them achieve academic success [29]. Undergraduate students’ perception of academic support is one of the major contributing factors to complete the course work successfully and a lack of support may be problematic for undergraduate students. Aslam et al., (2005) demonstrated that mentorship by lecturers play an important role in student research [15].

Developing positive attitudes in students towards research is a key element in current education system [30, 31]. Understanding the attitudes of students towards research is a first step to address the negative views of students towards research. These attitudes will help to describe the pattern of feelings, beliefs, and their reactions regarding research based on their past experience [32, 33]. Hence, the objective of this study was to measure pharmacy students’ anxiety towards research and how academic support, academic effort, attitude and their self-efficacy influence their research anxiety.

## Methods

### Ethical approval

The study was reviewed and approved by the International Medical University-Joint Committee, on Research and Ethics (BP I-01-12 (49) 2015). Before collecting the data, participants’ written consent was obtained. Confidentiality and anonymity were retained throughout this study.

### Sample frame

A cross-sectional study was conducted for 6 months from June to November, 2015, in International Medical University, Kuala Lumpur, one of the private universities in Malaysia. All final year pharmacy students in this university were invited to participate. All the students were informed that the survey will be anonymous and recorded responses would be used for scientific purposes only. Though all students were invited to participate, a minimum sample size was needed to prove the significance of the study. Hence, using a Raosoft software calculation, a required sample size of 110 was demonstrated; power 80%, distribution of response 50%, with 95% confidence interval and a 5% margin of error. A total of 149 pharmacy students was approached, but the final participating number was 128 which was more than the required sample size to generalize the findings. Convenience sampling was done. The only exclusion criterion was disinterest in participating.

### Sampling procedure

International Medical University, Pharmacy students have 8 semesters in their total duration of 4 year course. During final year that is during their 7th semester a six month (24 weeks) research project is mandatory. So the current study targeted these pharmacy students in order to measure their anxiety towards research and how academic support, academic effort, attitude and their self-efficacy influence their anxiety towards research. The researchers of this study approached the pharmacy students during their almost end of semester 7 (week 16–20). These time durations were selected to collect the data as the students had more or less completed their research work by these weeks.

### Study tool

The questionnaire was in English which had 48 items with six sections. The first section extracted demographic information of the participants with 5 questions in it. The second section, comprising 8 questions, evaluated the academic support of participants towards research. The third section evaluated the academic effort of participants towards research by assessing their responses to 7 questions. The fourth section, comprising 8 questions, evaluated the self-efficacy of participants towards research. The fifth section, comprising 10 questions assessed the attitude of participants towards research. The sixth section, comprising 10 questions, assessed the anxiety of participants towards research. All the responses, except demographic details were recorded using a 5 point Likert scale of agreement. A score of 1 was given to strongly disagree, 2 to disagree, 3 to Neutral, 4 to agree and 5 to strongly agree. Negatively worded statements were reverse coded.

### Validity and reliability of the study tool

A self-administered questionnaire was prepared based on relevant published studies [22, 24, 28, 29, 32]. An initial version of the questionnaire underwent face and content validity. Content validity was done by a panel of 3 subject experts and their opinion on the relativity and the significance of the questionnaire was considered [34]. Necessary adjustments were amended to the questionnaire based on the experts’ opinion prior to its administration to a small group of 10 students for a pilot study. A pilot study was done to confirm the reliability of the questionnaire. By using SPSS V.20 the internal consistency reliability was estimated by coefficient alpha index with reference to the Cronbach’s alpha value. Cronbach’s alpha is an important concept in the evaluation of questionnaires. It is mandatory that researchers should estimate this quantity to add validity and reliability to interpret their data. The acceptable values of alpha, ranging from 0.70 to 0.95 [35, 36]. In this study, Cronbach’s alpha was 0.70 for academic support, 0.72 for academic effort, 0.78 for self-efficacy, 0.72 for attitude and 0.74 for research anxiety sections. For the final analysis, the data of the pilot study were not used.

### Data analysis

Frequencies and percentages were presented by using descriptive analyses. The normality of the data was verified by using Kolmogorov-Smirnov test and the significant value were below 0.05 suggesting violations of the assumption of normality. As the data distribution was not normal, skewness of the data was analysed. While determining skewness values, if the calculated z value for skewness lie less than the critical values of ±1. 96 at 0.05 significance level, then the distribution of data is considered normal [37]. In the current study, the skewness tolerance value was 1.90 and hence the data were considered normally distributed. Mean scores for each parameter [38] (academic support, academic effort, self-efficacy, attitude and anxiety to research) was taken. Total mean scores of these parameters were used for inferential analysis. In order to examine the relationship between the students’ academic support, academic effort, self-efficacy, attitude and anxiety to research, a Spearman Rho test was employed. The absolute value 0.25 or above with p-value < 0.05 was measured as statistically significant at 95% confidence level. Outliers were investigated using Mahalanobis Distance which is 8.35 and it is within the limit.

## Results

Of the total pharmacy student population of 149, 128 pharmacy students participated in this study, giving a response rate of 85.9%. The majority were females (74.2%). The demographic profiles of the respondents are shown in Table 1.

**Table 1:** Demographic information of respondents.

Socio-Demographic Characteristic	Frequency	Percentage (100%)
**Gender**		
Male	33	25.8
Female	95	74.2
Total	128	100
**Race**		
Malay	2	1.6
Chinese	116	90.6
Indian	8	6.2
Others	2	1.6
Total	128	100
**Do you have a previous degree?**		
Yes	2	1.6
No	126	98.4
Total	128	100
**Do you have previous work experience?**		
Yes	92	71.9
No	36	28.1
Total	128	100
**Involved in any research activity before?**		
Yes	31	24.2
No	97	75.8
**Total**	**128**	**100**

Table 2 presented students’ view of academic support towards research. Participants agreed that their lecturers help in research ($\bar{\text{x}}$ = 4.11; SD = 0.79) and their friends give emotional support in the research activities when needed ($\bar{\text{x}}$ = 3.96, SD = 0.80). These two statements remained on the top two with higher mean scores for academic support.

**Table 2:** Academic support of participants towards research.

Academic support	Mean	SD
My friends/peer really try to help me in research	3.96	0.72
My lecturers really try to help me in research	4.11	0.79
I get the emotional help and support I need from my friends/peers	4.05	0.80
I get the emotional help and support I need from my lecturers	3.73	0.90
I can talk about my problems with my friends/peers	4.02	0.70
I can talk about my problems with my lecturers	3.75	0.86
My friend/peer is willing to help me make decisions	3.88	0.77
My lecturer is willing to help me make decisions	3.88	0.78
**Total mean score**	**31.43**	**6.31**

Table 3 showed academic effort of participants towards research. Participants agreed that they read the research literature more than once in order to gain a full understanding of the research ($\bar{\text{x}}$ = 4.01, SD = 0.77), and “least keen in attending any research methods and statistics courses conducted” ($\bar{\text{x}}$ = 3.29, SD = 1.01). These two statements explained their academic effort to understand research by themselves.

**Table 3:** Academic effort of participants towards research.

Academic effort	Mean	SD
Apart from other course assignments, I also can do extra exercises related to research and statistics.	3.44	0.91
Apart from reading the lecture notes, I also read extra readings/information related to research and statistics.	3.50	1.06
I work hard to complete the research.	3.98	0.67
I study hard and prepared well for research methods and statistics.	3.66	0.77
In order to get full understanding on research, I read the research literatures more than once.	4.01	0.77
I am keen to attend research methods and statistics courses conducted.	3.29	1.01
I paid close attention to research methods and statistics courses.	3.58	0.85
**Total mean score**	**25.46**	**3.95**

Table 4 showed participants’ self-efficacy towards research. Participants indicated that they could gather literature on their research work from different sources ($\bar{\text{x}}$ = 3.78, SD = 0.70). This is an indication of their ability to collect the research related information from the available resources. However, the statement ‘I am able to understand the statistics formulas ($\bar{\text{x}}$ = 3.04, SD = 0.90) got less score reflected their difficulty in understanding statistics.

**Table 4:** Self-efficacy of participants towards research.

Self-efficacy of participants	Mean	SD
I am able to learn research methodology and data analysis	3.72	0.64
I am interested to participate in research related courses	3.55	0.84
I am able to understand about research methodology and data analysis.	3.74	0.66
I am able to understand the statistics formulas	3.04	0.90
I am able to explain and tutor other students about research	3.28	0.86
I can do research methods and data analysis even if it is the hardest work	3.27	0.78
I can figure out difficult part in research	3.23	0.84
I am able to gather information regarding research from different sources	3.78	0.70
**Total mean score**	**27.72**	**6.27**

The attitude of participants towards research is shown in Table 5. Participants felt that they are under stress while doing research ($\bar{\text{x}}$ = 4.29, SD = 0.90) and the mean value for the statement “research is a required part of their professional career” was low ($\bar{\text{x}}$ = 2.38, SD = 0.72). The participants’ thinking about the importance of research in their professional career might influence their interest and this may be a reason for their stress during research.

**Table 5:** Attitude of participants towards research.

Students’ attitude	Mean	SD
I like research.	3.47	0.72
I enjoy doing research.	3.42	0.79
I am confident when I have to deal with research.	3.13	0.80
I am under stress while doing research.	4.29	0.90
I can learn from research.	3.88	0.70
I find it difficult to understand research concepts.	2.54	0.86
I find it difficult to understand research methods.	3.30	0.77
I find statistics formulas are easy to understand.	3.35	0.77
Research is a required part of my professional career.	2.38	0.72
Research skills make me more employable.	3.89	0.79
**Total mean score**	**33.66**	**0.76**

Table 6 shows participants’ anxiety towards research. Participants said that they struggle with writing research reports, or avoid them as long as they can ($\bar{\text{x}}$ = 3.94, SD = 1.16). During data analysis, participants felt that they were doing badly or that they may fail in their research projects ($\bar{\text{x}}$ = 3.89, SD = 1.05). Their lack of research writing skills and less understanding of statistical analysis may be the reasons.

**Table 6:** Anxiety of participants towards research.

Research Anxiety	Mean scores	SD
The closer I am to research work; the harder it is for me to concentrate on that.	3.45	0.88
When I perform research, I worry that I will not remember the research protocol.	3.04	1.03
During data analysis, I think that I am doing awful or that I may fail.	3.89	1.05
I lose focus on research and cannot remember important aspects.	3.45	0.84
Usually I remember what to do after the experiment is already over.	3.23	0.95
I worry so much before my performance that I am too worn out to do my best.	3.82	1.04
I feel out of sorts or not really myself when I take any research related tasks.	3.59	0.94
I find that my mind sometimes wanders when I am taking an important research task.	3.70	1.07
After completing a research project, I worry about whether I did well enough.	3.05	0.93
I struggle with writing research reports, or avoid them as long as I can.	3.94	1.16
**Total mean score**	**32.16**	**9.87**

Correlation and multiple regression analyses were conducted to examine the relationship between students’ research anxiety and predictor variables (students’ academic support, academic effort, self-efficacy and attitude) (Table 7).

**Table 7:** Descriptive statistics, predictor variable correlations, multiple regression results.

	Mean	SD	Academic support	Academic effort	Self-efficacy	Attitude	Research Anxiety	b	Standard error	Beta
Academic support	31.43	6.31	1	-	-	-	-	-0.399	0.033	-0.458***
Academic effort	25.46	3.95	0.28	1	-	-	-	-0.379	0.033	-0.405**
Self-efficacy	27.72	6.27	0.54	0.59*	1	-	-	0.363	0.238	0.729
Attitude	33.66	0.76	0.42	0.64	0.48	1	-	-	-	-
Research Anxiety	29.56	9.87	-0.88**	-0.60*	-0.65*	0.39	1	-	-	-

* p <0.05,

** p < .01,

***p < .001

The predictor variables’ scores (students’ academic support, self-efficacy, academic effort) was negatively and significantly correlated with the criterion variable (research anxiety) except one predictor variable (attitude of students towards research). There was a strong negative correlation between academic support ($\bar{\text{x}}$ = 31.43; SD = 6.31) and research anxiety ($\bar{\text{x}}$ = 29.56; SD = 9.87), r (126) = -0.88, p = 0.003; students’ self-efficacy towards research ($\bar{\text{x}}$ = 27.72; SD = 6.27) and research anxiety ($\bar{\text{x}}$ = 29.56; SD = 9.87), r (126) = -0.65, p = 0.004. There was a moderate negative correlation between students’ academic effort towards research ($\bar{\text{x}}$ = 25.46; SD = 3.95) and research anxiety ($\bar{\text{x}}$ = 29.56; SD = 9.87), r (126) = -0.60, p = 0.02. There was no correlation between students’ attitude towards research and research anxiety r (126) = 0.39, p = 0.41. Hence, this variable ‘students’ attitude’ was not included in multiple regression.

Before conducting multiple regressions, the relationship among the predictor variables were checked to rule out multicollinearity. Visual examination using scatter plots showed no significant correction between academic support and academic effort. Similarly, there was no correlation between academic support and self-efficacy. However, there was a mild correlation between academic effort and self-efficacy are (126) = 0.29, p = 0.04. Since the variance inflation factor was 2.2 and the tolerance value was 0.92, multicollinearity was neglected [39].

The stepwise multiple regression with three potential predictors was analysed. Only two variables (academic support and academic effort) were significantly affected the research anxiety. In model 1, only with academic support variable, 57.6% of the research anxiety decrease is explained F (1, 8.317) = 95.69, R^2^ = 0.576, p < 0.005. In model 2, academic effort along with academic support were the significant predictors with F (2, 7.239) = 80.21, R^2^ = 0.798, p < 0.001. Model 2 was selected in this study as the R^2^ value was higher than (79.8% explained variance) model 1. In model 2, there was a further decrease in research anxiety with significant R^2^ change of 22.2% variance. The non-significant contributor (self-efficacy) was excluded automatically by the SPSS. Outlier was investigated using Mahalanobis Distance which is 8.35 and it is within the limit.

When individual coefficients were analysed to determine the strongest predictor, academic support with the standardized coefficient beta value of -0.458 was the best predictor. When analysed for the shared and unique contribution of the predictors, academic support has shared (0.712)^2^ = 50.6%, unique (0.633)^2^ = 40% to the dependent variable (research anxiety). Academic effort has shared (0.702)^2^ = 49.2%, unique (0.532)^2^ = 28.3% contribution to the dependent variable (research anxiety).

## Discussion

The results indicated that, lecturers provided academic support and their friends/peers provided emotional and other support when the participants were doing research. Apart from the quality of curriculum and instructions in the classroom, effective assistance in learning and academic advice also help the students to perform in academics and research. Research focused mentoring relationships will help the students to increase their interest in research [40]. A peer-support strategy at the undergraduate level provide social and emotional support for the students [41]. Additionally, the lecturer’s involvement as a research supervisor during undergraduate research has an impact on the successful completion of students’ research projects.

The total mean score for academic effort indicated that students showed much interest to read literature, i.e., published research articles to understand their research, despite their lesser interest in attending classes on research methods and statistics courses. Studies have reported that hands-on activities may help or motivate students to learn research and statistics [42, 43].

The participants’ ability to learn and understand research methods and their capability of gathering information from research articles using various sources are the indicators of their self-efficacy towards research. The confidence level of students to perform research activities is directly proportional to their self-efficacy towards research methods and statistics [44]. The participants mentioned that some parts of the research, such as statistical formulae, are difficult to understand. The issue of understanding statistical analysis may be a challenge for the students at undergraduate level and this issue can be overcome by learning. The majority of the pharmacy related research use methods to quantify phenomena, associations or relationships between variables. Therefore, understanding various statistical procedures employed is important to interpret the findings [45].

The participants’ attitude towards research indicated their positive outlook on research. Studies have shown that, students are more likely to put effort into studying research methods and statistics when they possess a positive attitude towards the subject [46, 32]. Most students were reported that they are under stress, while doing research. Non-availability of resources to cope with a perceived situation can result in stress of an individual’s perception [47]. A key mechanism for educational institutions to minimise student stress is to provide appropriate resources on research tools and materials. Most participants mentioned that research is not an essential component of their professional career. Therefore, structured programmes and strategies focusing to provide positive views on research must be developed and implemented in curricula.

The participants are anxious about research and findings. Anxiety in any learning activity may affect students’ achievement [48]. One of our earlier studies found that an excessive demand of writing research related documents was one of the reasons to reject academic career [49]. As the students struggle with writing research related documents, it may trigger their anxiety towards the course and they may find the course difficult. This may lead to a negative attitude towards research. Hu et al., (2007), reported that students’ research experiences have a significant effect on their learning process [50]. Writing research proposals and grant applications are not part of the undergraduate student's repertoire in their curriculum [49]. This may also lead them to be anxious about research. Academic anxiety is experienced by students of all academic levels at some time [51, 52]. Those students who performed well in class also have suffered from test anxiety at some time [53]. Peers and lecturers can help those students who have anxiety towards research by helping them understand the learning process. Studies have shown that academic support, academic effort, and self-efficacy are significantly and negatively correlated with research anxiety [45, 54–57]. However, in this study, self-efficacy did not moderate the effect of the research anxiety. A similar report was published by Barrows et al., who reported the association between students’ anxiety and their lower academic performance [58].

Based on the results from this study, the academic support and academic effort were the significant contributors to reduce research anxiety. Academic performance of students has been found to be improved for those who seek and receive academic support. Academic support helps the students to develop higher self-expectations, a greater sense of self-perceived control of academic outcomes for future academic success [59]. At the same time, increasing academic effort on a task by students may partially or completely divert the effects of anxiety [60, 61].

The finding also showed that there is no relationship between pharmacy students’ attitude towards research and their research anxiety is contrary to a study by Kritikos et al., (2015) which showed a positive relationship between student attitude and anxiety towards research [62]. The exact reason for this difference in results is unknown. A possible reason for this contradiction may be due to the current scenario in Malaysia, where the focus of undergraduate pharmacy education is more towards pharmacy practice, and research is not a primary career choice [63]. The fresh graduates prefer to get a job in hospitals and community pharmacy where they will able to practice. The willingness of fresh graduate to enter into research holds the key to the future pharmacy research initiatives in Malaysia. While pharmacy research is normally associated with industry and academia, a sound pharmacy education system which emphasis on research and skills needed in research as well as partnership in research will surely bring forth tremendous progress to the pharmacy profession in Malaysia.

## Limitations

As with all studies relying on voluntary participation, results can be biased. As a self-administered questionnaire was used in this study, social desirability bias can occur in the results which is inevitable.

## Conclusions

This report reveals that academic support, academic effort, and self-efficacy are negatively correlated with pharmacy students’ research anxiety. Academic support was the predominant factor to reduce research anxiety among pharmacy students. Therefore, implementing a pharmacy curriculum which emphasis on research and skills needed in research may bring change in students’ attitude towards research and their self-efficacy to deal with their anxiety towards research.
